# Implementing whole blood TBI point-of-care testing in Level 1 trauma centers: protocol for emergency department implementation for TBI (EDI for TBI) hybrid type II effectiveness–implementation trial

**DOI:** 10.3389/frhs.2026.1891721

**Published:** 2026-07-28

**Authors:** Shawn Eagle, Molly McNett, Debbie Madhok, Jeffrey Bazarian, David Barton, Kathryn Edelman, Jane Sharpless, Gigi Sugar, Christine Gotthardt, Alan Wu, Victoria Zhang, Nam Tran, Sarah Degener, Kian Merchant-Borna, Geoffrey Manley, David Okonkwo

**Affiliations:** 1 Department of Neurological Surgery, University of Pittsburgh, Pittsburgh, PA, United States; 2 College of Nursing, Ohio State University, Columbus, OH, United States; 3 Department of Emergency Medicine, University of California San Francisco, San Francisco, CA, United States; 4 Department of Emergency Medicine, University of Rochester, Rochester, NY, United States; 5 Department of Emergency Medicine, Florida Atlantic University, Boca Raton, FL, United States; 6 Department of Neurological Surgery, University of California San Francisco, San Francisco, CA, United States; 7 Department of Laboratory Medicine, University of California San Francisco, San Francisco, CA, United States; 8 Department of Pathology and Laboratory Medicine, University of Rochester Medical Center, Rochester, NY, United States; 9 Department of Pathology, University of Pittsburgh, Pittsburgh, PA, United States

**Keywords:** blood biomarkers, computed tomography scans, emergency department, GFAP, point-of-care, traumatic brain injury

## Abstract

**Introduction:**

Technological advances have enabled rapid screening for traumatic brain injury (TBI) at the point of care (POC), with devices approved by the United States Food and Drug Administration (FDA), in part to address throughput and resource allocation challenges in emergency departments (EDs). This point-of-care test quantifies the concentration of two biological markers of brain injury in whole blood after venipuncture.

**Methods:**

The objective of this study is to identify determinants for routine use of POC whole blood TBI test and to evaluate its impact on clinical and health system outcomes across three academic Level 1 trauma centers with accompanying EDs from three distinct geographical regions of the United States. We will utilize an effectiveness–implementation hybrid type II approach for this study involving 280 patients with Glasgow Coma Scale (GCS) scores of 13–15 (i.e., *“*mild*”* TBI).

**Results:**

Data will be collected (*n* = 140) using the POC whole blood TBI test, while simultaneously evaluating determinants of use to inform future widespread implementation efforts. An implementation blueprint will be developed and pilot tested at each site, while concurrently enrolling the final 140 patients with GCS 13–15.

**Discussion:**

Our goal is to provide evidence that use of the POC whole blood TBI test can reliably and safely improve clinical practice in EDs by reducing rates of unnecessary radiation exposure, shortening time spent in the emergency department, and demonstrating the efficacy of an implementation blueprint that can facilitate uptake of this test at similar Level 1 trauma center emergency departments.

**Clinical Trial Registration:**

[ClinicalTrials.gov], identifier [NCT07373509]

## Background

Approximately 5 million patients will be evaluated annually in United States emergency departments (EDs) for traumatic brain injury (TBI), and over 80% will undergo head computed tomography (CT) scans to identify potential intracranial pathology (1). Despite the high rate of head CT scans, <10% will be positive for intracranial pathology. Further, more than 90% of those evaluated will have a *“*mild” TBI [mTBI; categorized as Glasgow Coma Scale (GCS) 13–15], which is a type of brain injury associated with even lower rates of positive head CT scans (1). This clinical workflow results in millions of patients being unnecessarily exposed to radiation and increased ED visit costs. Overuse of CT scans in general contributes to ED overcrowding across the country (2). This issue is pervasive across both civilian and military EDs (3), resulting in negative impacts for individual and unit level readiness, particularly when resources are diverted away from additional emergency situations. There is also a significant financial impact due to these delays and overcrowding, as ED visits are more costly and resource-intensive than primary care appointments (4).

Technological advances have led to the development of rapid screening for TBI, approved by the United States Food and Drug Administration (FDA), in part to address throughput and resource allocation issues in EDs. The i-STAT Alinity TBI point-of-care test quantifies the concentration of two biological markers of brain injury in whole blood after venipuncture: glial fibrillary acidic protein (GFAP) and ubiquitin c-terminal hydrolase-L1 (UCH-L1) (5). GFAP is a filament protein found in astrocytes that provides structural support and stability to the central nervous system. UCH-L1 is an enzyme that recycles ubiquitin, an important regulatory protein. This test was FDA approved in 2024 as a Clinical Laboratory Improvement Amendment (CLIA) moderate complexity test for ruling out the need for a head CT scan when both biomarker values are below clinical cutoffs, achieving a nearly 100% negative predictive value (6). The test requires 15 min to deliver a result and does not require sample centrifugation to obtain plasma; instead, the test can be performed at the point of care (POC) using a small test cartridge and the Alinity iSTAT handheld reader.

### Preliminary work

In a preliminary study conducted at a Level 1 trauma center ED with 104 patients presenting with suspected mTBI (7), we found that the POC whole blood TBI test delivered results 2.5 h faster than a head CT scan (even when using the original plasma version of the test, which requires laboratory processing). As most EDs do not have a centrifuge, the use of plasma as the testing matrix requires transport of the sample to the central clinical laboratory for receipt, accessioning, centrifugation, and the delivery to the testing area. With the new POC whole blood TBI test, we anticipate additional time savings of 45 min due to not having to send the sample to the central laboratory. Emergency medicine physicians were surveyed about their decision to obtain head CT scans despite negative values for both biomarkers, indicating unfamiliarity with the POC whole blood TBI test, familiarity with the CT scan, and the perspective that the CT scans are more sensitive/specific. All patients received a head CT scan, regardless of the POC whole blood test result. Overall, nearly one in five patients could have avoided a head CT scan (due to a negative POC whole blood TBI test) in this sample (7).

### Current work

Despite receiving FDA clearance, recognition in national news campaigns, and electronic distribution of test information, ED providers do not routinely utilize this POC whole blood TBI test when evaluating patients with suspected mTBI or determining the need for CT scans. Others have identified additional barriers to routine use. These include entrenchment of CT scans as standard of care, narrow indication of clinical use (FDA approval limited to mTBI patients to rule out the need for a head CT scan), no recommendation yet in clinical guidelines, integration concerns with electronic health records, absence of a current procedural terminology code, and lack of education/awareness about the test and its cost-effectiveness (8–10). To address identified barriers, additional data on clinical effectiveness are needed to determine impact on patient and health system outcomes. In particular, there are no data to indicate other potential uses of the test to predict clinically relevant outcomes beyond ruling out head CT, such as ED length of stay and the incidence of return ED visits. In parallel, there is also an ongoing need to better understand contextual barriers to routine use among frontline clinicians. Therefore, the objective of this study is to identify determinants for routine use of the POC whole blood TBI test within ED settings and to evaluate impact on clinical and health system outcomes.

Each phase of the study will aim to do the following:

Phase 1: Evaluate determinants for routine use of the POC whole blood TBI test by assessing acceptability, adoption, appropriateness, and feasibility of the test at the organizational and provider level for each site and determine how these factors interplay with patient perspectives and knowledge to influence implementation of the test.
Phase 2: Evaluate the effectiveness of the POC whole blood TBI test in determining the need for a head CT scan in approximately 140 patients with a Glasgow Coma Scale (GCS) scores of 13–15 in three Level 1 trauma center EDs (∼47 per site) while assessing secondary outcomes with clinical implications (i.e., reduction in ED length of stay and predictive utility for ED revisits for the same injury).
Phase 3: Develop a site-specific implementation blueprint for use of the POC whole blood TBI test in determining the need for a head CT scan.
Phase 4: Pilot test the implementation blueprint in approximately 140 patients with GCS scores of 13–15 in the ED (∼47 per site).

## Methods

**Figure 1:**
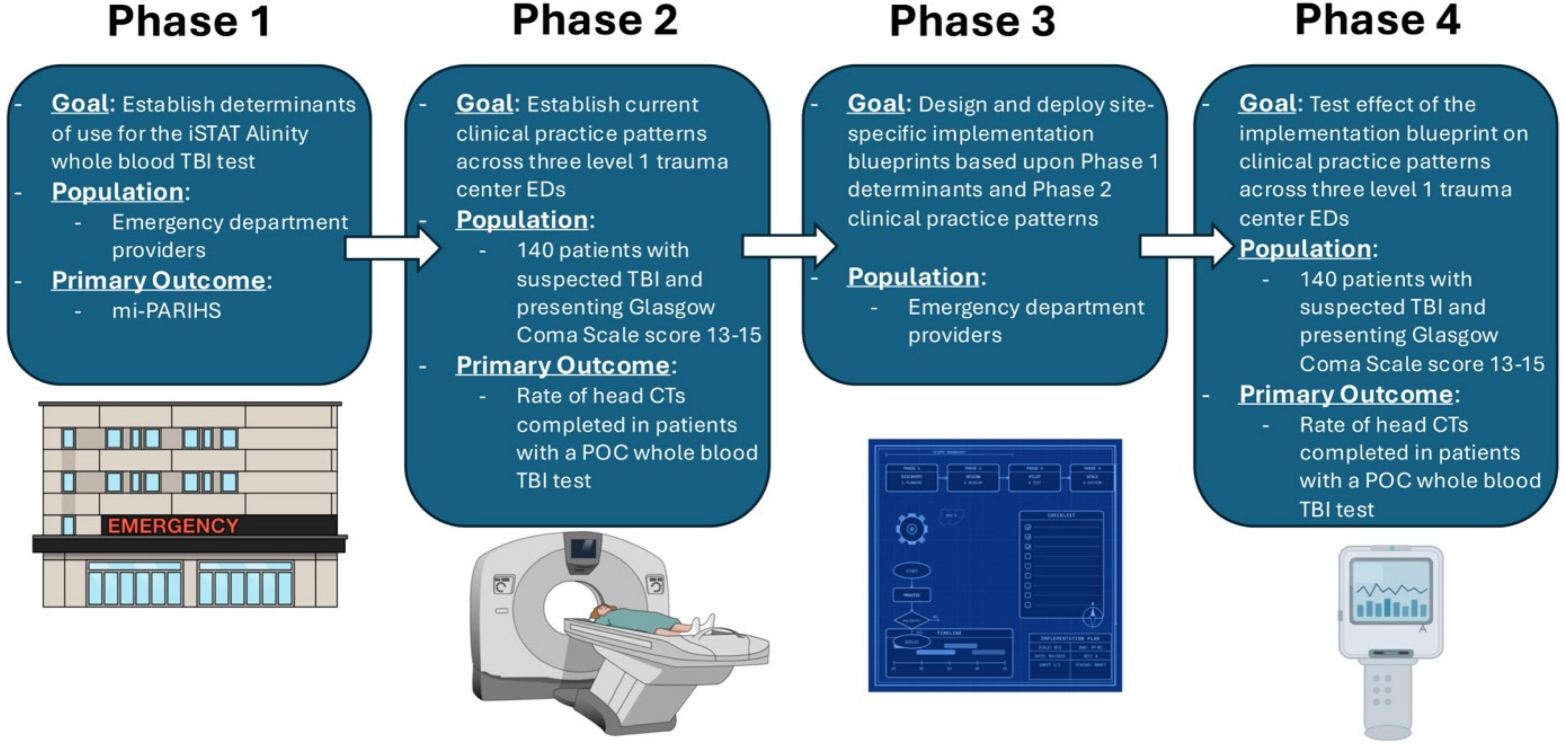
Study protocol in phases.

Figure 1 illustrates the four phases of the study, including the goal, study population, and primary outcome measure. This study aims to enroll 280 patients with acute GCS scores of 13–15 across three sites (Phase 2 = 140, Phase 4 = 140). The three enrolling sites represent academic Level 1 trauma centers with accompanying EDs from three distinct geographical regions of the United States. Target enrollment for each site includes 47 acute ED patients in Phases 2 and 4.

### Patient participants

The study team will identify potential participants with suspected TBI according to approved local processes. Eligibility criteria may be reviewed through electronic medical records and patient interviews. Eligible patients may be approached to discuss participation. Enrolled patients will be asked to complete a brief battery of questionnaires about their injury and knowledge about concussion and radiation.

### Eligibility criteria

Inclusion criteria for patients are as follows: (1) age 18 or older, (2) presenting to an enrolling ED with arrival GCS 13–15 following reported blunt head impact, (3) presenting to the ED within 24 h of injury, and (4) meets Department of Defense definition of mTBI (a traumatically induced physiological disruption of brain function after external force, indicated by new onset or worsening of at least one of the following clinical signs: any alteration of mental status, any period of loss or decreased level of consciousness, or any loss of memory for events immediately before or after the injury). Exclusion criteria for patients are as follows: (1) treating ED provider does not determine need for a head CT, (2) inability to obtain results for POC whole blood TBI test within 24 h of injury, (3) preexisting neurologic condition, (4) contraindication or inability to complete blood draw, (5) significant polytrauma that, in the opinion of the principal investigators, could impact peripheral GFAP levels or require a full-body scan, (6) history of penetrating TBI, (7) history of neurosurgical intervention for previous TBI, (8) penetrating TBI for current injury, (9) any history of brain surgery, (10) any history of brain tumor, and (11) patients on psychiatric hold.

### Funding and ethics approval

This study received the Health Sciences Research Award from the Congressionally Directed Medical Research Program's Traumatic Brain Injury and Psychological Health Research Portfolio (No. HT94252510275). The study received ethical approval from the University of Pittsburgh's Institutional Review Board on 24 November 2025 (No. STUDY25010137) in accordance with the Declaration of Helsinki. The study's protocol was registered on ClinicalTrials.gov on 4 February 2026 (No. NCT07373509) prior to the initiation of study procedures. All human subjects will provide informed consent prior to completing study procedures.

### Study design

We will utilize an effectiveness–implementation hybrid type II approach for this study involving 280 patients with GCS scores of 13–15 presenting to three civilian EDs (11). Study phases and progression are illustrated in Figure 1. In Phase 1, we will evaluate determinants for use to inform future widespread implementation efforts. In Phase 2, we will collect clinical effectiveness data using the whole blood TBI test. In Phase 3, we will develop an implementation blueprint for each site and disseminate to local stakeholders. In Phase 4, we will evaluate if clinical practice patterns change at each site in a separate cohort of 140 patients with GCS scores of 13–15.

### Whole blood TBI test

POC testing will be conducted using the iSTAT Alinity whole blood TBI cartridge and point-of-care analyzer (Abbott Laboratories, Chicago, IL, USA). The POC whole blood TBI test will be performed at each enrolling site within the FDA-approved indication and will be operated by appropriately trained personnel (6). Quality control and product validation will be performed according to manufacturer and CLIA requirements. A single venipuncture will be performed in the ED to obtain ∼3 mL of whole blood to be used for testing.

### Community advisory board

A diverse community advisory board (CAB) of stakeholders related to the implementation of the POC whole blood TBI test in the ED will be assembled to assist in Phases 3 and 4. The CAB will be assembled by the University of Pittsburgh's Clinical Translational Science Institute and will include representatives from each enrolling institution to provide site-specific context. Members of the CAB will include (from each site) recent acute mTBI patients in the ED, hospital administrators, ED physicians, ED staff (e.g., medical assistants), ED nurses, and the team's implementation scientist. The CAB will be assembled two times per year during the study period for a virtual meeting. Objectives of the initial meetings will be to brief the CAB on the final study design, outcomes (and what they mean), and objectives for conducting the study. Input from the CAB will be integrated into study procedures prior to the initiation of the study. Objectives of the subsequent meetings will be to brief the CAB on findings from Phases 1 and 2, and to discuss determinants (in their view) of successful implementation of the test.

#### Phase 1

The goal of Phase 1 is to establish determinants of use for the POC whole blood TBI test for each enrolling site's ED. To accomplish this goal, we will survey ED healthcare providers to identify determinants of routine use, decision-making processes, and perspectives on acceptability, appropriateness, adoption, and fidelity.

To evaluate determinants of routine use, the Mobilizing Implementation of iPARIHS Facilitation Planning tool (Mi-PARIHS) will be administered as the primary outcome measure for this phase. The Mi-PARIHS is a 34-item tool which supports users in their assessment of the innovation, context and recipient constructs, development of a tailored facilitation plan, and repeated use over time to evaluate the effectiveness of facilitation strategies. Each question is rated on a five-item Likert scale to identify barriers and facilitators/enablers for the innovation being evaluated, where −2 indicates a barrier and +2 indicates a facilitator for that aspect of implementation. In alignment with the i-PARIHS framework, domain scores are summed across innovation, recipients, and context items where higher scores indicate more facilitators/enablers and lower scores indicate more barriers.

The Decisional Conflict Scale (DCS) will be used to assess factors influencing the decision-making processes of providers when deciding between use of the POC whole blood TBI test and CT scanning (12). The DCS measures five dimensions of decision-making, evaluating uncertainty, lack of information, unclear values, lack of support, or ineffective decision-making. The acceptability of intervention measure (AIM) (13) is a five-item measure evaluating the perception among implementation stakeholders that a given practice is agreeable or satisfactory. This tool will assess providers’ perspectives on how acceptable the POC whole blood TBI test is for assessing need for CT and determining the plan of care for patients presenting with mTBI. The AIM has excellent structural and known-groups validity as well as test–retest reliability [intraclass correlation coefficient (ICC) = 0.82] (13). The Intervention Appropriateness Measure (IAM) will also be administered to assess baseline perceptions of the appropriateness of the POC whole blood TBI test (13). The IAM is a five-item measure evaluating the perceived relevance of the test for a given setting. The IAM has excellent structural and known-groups validity as well as test–retest reliability (ICC = 0.94) (13). Adoption will be evaluated by the Adoption of Information Technology Innovation (AITI) (14), a 25-item instrument designed to study adoption of new technologies. The scale has excellent reliability and validity metrics (15). The Williams Intention to Adopt Measure (WIAM) will also be used to evaluate adoption (16). The WIAM will be adapted to the current study to understand the willingness of ED providers to adopt the POC whole blood TBI test. This is a five-item measure where the provider will answer if they have spoken with colleagues about experiences with the POC whole blood TBI test; whether they intend to use the POC whole blood TBI test at each visit; whether they have attended trainings, workshops, or other learning sessions focused on the POC whole blood TBI test; and whether they have searched the literature about point-of-care blood tests for TBI for use with patients. The provider will be asked to rate their intended use of the POC whole blood TBI test across the next 10 patients seen in the ED. The WIAM uses a seven-point scale, ranging from 1 (strongly disagree) to 7 (strongly agree), except for the final question, which ranges from 0 to 10. The WIAM has some of the best psychometric properties among adoption measures (16). Lastly, feasibility will be assessed at baseline using the Feasibility of Intervention Measure (FIM) (13). The FIM is a five-item measure which evaluates the extent to which an assessment can be successfully carried out within a given clinical setting. The FIM has excellent structural and known-groups validity, as well as test–retest reliability (ICC = 0.87) (13).

#### Phase 2

The goal of Phase 2 is to enroll approximately 140 patients across the three enrolling site EDs to establish current clinical practice patterns with regard to obtaining head CTs for patients who meet the study's inclusion/exclusion criteria. Prior to initiating patient enrollment, site principal investigators will brief emergency medicine and trauma faculty about the research study, the POC whole blood TBI test, and how to interpret the test results. The intent of this phase is to establish early stakeholder engagement and obtain feedback from frontline clinicians that will inform development of the implementation blueprint in Phase 4.

Timeline of participant outcome assessments can be viewed in Table 1. The primary endpoint of this phase is the rate of head CTs completed in enrolled patients. The secondary endpoint of this phase is the rate of head CTs completed in enrolled patients with negative POC whole blood test results. These primary and secondary endpoints were selected to match the FDA's framing of clinical benefit and to be directly comparable to ongoing and completed studies evaluating utility of blood biomarkers for CT utilization in the ED (6, 17–19). Additional secondary outcomes include ED length of stay (in minutes), return to ED for the same injury within 2 weeks of first ED presentation (yes/no), Military Acute Concussion Evaluation-2 (MACE-2) results, Neurobehavioral Symptom Inventory (NSI), and two customized surveys about knowledge of mTBI and knowledge of the effects of radiation exposure in healthcare settings. ED length of stay can be extracted from the electronic medical record by research personnel, as presentation and discharge from the ED are timestamped. The MACE-2 is a multimodal tool which assesses clinical signs of TBI, incident description, signs, symptoms, medical history, a brief cognitive exam, neurological exam, and modified Vestibular Ocular Motor Screening (VOMS). The MACE-2 takes approximately 15 min to complete. The NSI is a 22-item scale of TBI symptoms since the time of injury.

**Table 1:** Timeline of patient participant outcome assessments.

Outcome	Enrollment	Two-week follow-up
Military Acute Concussion Evaluation—2	X	
Neurobehavioral Symptom Inventory	X	X
Traumatic brain injury knowledge	X	
Radiation exposure knowledge	X	
Quality of Life After Brain Injury Overall Scale		X
Follow-up care survey		X

Patients will also receive a series of surveys to complete remotely approximately 2 weeks after the initial visit. Patients will complete the NSI, the Quality of Life After Brain Injury Overall Scale (QOLIBRI-OS), and a brief custom survey about follow-up clinical care. The QOLIBRI-OS is a 6-item tool evaluating how a participant's brain injury has impacted their health-related quality of life.

#### Phase 3

The goal of Phase 3 is to design and deploy site-specific implementation blueprints based on determinants of use identified in Phase 1 and stakeholder feedback and practice patterns from Phase 2. The i-PARIHS framework will be used to select implementation strategies that will address identified facilitators and barriers from the Mi-PARIHS assessment in Phase 1.

### Implementation strategies

The purpose of developing an implementation blueprint is to provide a guide for stakeholders and providers to enhance readiness and support the scale-up of new practices across different sites (20). As such, development of an implementation blueprint is a complex, dynamic, and collaborative process that engages members across three primary categories: (1) those responsible for implementing the new practice (i.e., providers, supervisors); (2) those who have a role in supporting the new practice and/or removing barriers to its implementation (i.e., providers, supervisors, clinical practice managers); and (3) those who would benefit from implementing the new practice (i.e., patients, providers) (15, 21). The CAB will be convened to operationalize the strategies specific to the local context and contribute to refining the implementation blueprint to maximize site-specific success. Implementation strategies for the blueprint will be selected based on contextual determinants identified in Phase 1 using the i-PARIHS framework (22) and the Implementation Strategy Supplemental Tool (23). A written implementation blueprint document will be developed with operational details for each implementation strategy using the Action, Actor, Context, Target, Time (AACTT) framework (24) to identify the setting, specific action steps, persons responsible, and timeframe to enhance replicability of the blueprint in future investigations across a variety of sites (15). The finalized blueprint will include a tailored manual of procedures (MOP) to support implementation of the practice change. This MOP will include the local leadership and stakeholder teams, identified barriers and facilitators to implementation, recommended implementation strategies, and specific action items and team activities required to support implementation. Activities to support implementation will be displayed using the Implementation Research Logic Model (25) and will include a phased timeline that aligns with common phases of implementation and incremental outcome metrics (26). The implementation blueprint will be deployed at each site prior to initiating Phase 4. Following study completion, the implementation blueprints will be published open access alongside the primary publications to enhance replicability.

#### Phase 4

The goal of Phase 4 is to test the effect of the implementation blueprint on clinical practice patterns with regard to obtaining head CTs for patients who meet the study's inclusion/exclusion criteria across the three enrolling sites. Approximately 140 patients with presenting GCS scores between 13 and 15 will be enrolled across the three sites. The primary endpoint of Phase 4 (to be compared with the outcome of Phase 2) is the rate of head CTs completed in enrolled patients. The secondary endpoint of this phase is the rate of head CTs completed in enrolled patients with negative POC whole blood test results. We will also evaluate if the implementation blueprint has impacted baseline perceptions of facilitators and barriers and related implementation outcomes (i.e., adoption, appropriateness, acceptability, decisions, and feasibility) has shifted provider's perspectives on the test by having those who completed Phase 1 surveys (e.g., Mi-PARIHS) complete the surveys again.

### Sample size

#### Power analysis

The primary endpoint of the overall study is to evaluate the effect of the implementation blueprint by comparing the proportion of patients receiving head CTs in the first patient cohort (enrolled in Phase 2) with the second patient cohort (enrolled in Phase 4). Given that 100% of patients in our pilot study received a head CT scan (regardless of POC whole blood test results), we expect the proportion of head CT scans to be high for Phase 2 enrollments. We hypothesize that a 20% reduction of head CT scans in Phase 4 would be possible after the implementation of the blueprint, representing a clinically meaningful reduction.

A power analysis was conducted using a two-tailed Fisher's exact test for two independent groups, using the proportions of 95% for Phase 2 and 75% for Phase 4, an alpha of 0.01, and power of 0.99. The analysis yielded a sample size of *n* = 140 in Phase 2 and *n* = 140 in Phase 4, with a total of 280 participants with mild TBI. G*Power (version 3.1.9.6) was used for the power analysis.

### Data management and analysis

Research data will be entered into a REDCap database managed by the University of Pittsburgh. Direct personal identifiers will be stored separately in REDCap and replaced with unique identification numbers for the purpose of protecting the identity of the source(s); the original identifiers will be retained in such a way that they can be traced back to the source(s) by someone with the linkage code. The linkage code will have restricted access to approved study team members at each participating site and will not be available to any external entity. To further protect against risk to confidentiality, any information with direct identifiers (i.e., names or other identifying information) will be kept separately and accessible only to approved research team members.

### Statistical analysis

Normality will be assessed for all outcomes, and appropriate transformations will be made to ensure that statistical assumptions are met prior to conducting all analyses. Statistical significance for these evaluations will be set at *p* < 0.05, and Bonferroni corrections will be applied to all analyses for multiple comparisons.

#### Phase 1

For Phase 1, descriptive statistics will be calculated for POC whole blood TBI test results, including the rates of elevated vs. non-elevated values, rates of CT scans ordered, and rates of return ED visits for the same injury within 2 weeks in the first group of 140 ED patient participants. Whether or not the patient returned will be self-reported at the 2-week follow-up survey sent to the participants. ED length of stay (in minutes) will be compared between patients who only received the POC whole blood TBI test and those who received the POC whole blood TBI test and a head CT scan using an analysis of covariance model, including enrollment site as a covariate. This analysis will be conducted only for participants with a negative POC whole blood TBI test to control for clinical severity of the injury. An exploratory analysis using receiver operating characteristic area under the curve (AUC) will be conducted to differentiate patients who return to the ED for evaluation of the same injury within 2 weeks from those who do not, using whole blood GFAP and UCH-L1 as predictors. Approximately 5% of patients diagnosed with TBI and GCS scores of 13–15 in the ED return to the ED within 72 h of discharge for TBI evaluation (27). The expected event rate would yield seven returns to the ED for the Phase 2 cohort of *n* = 140, which is lower than the standard expectation of 10 events per predictor. Statistical significance for these evaluations will be set to *p* < 0.05.

#### Phase 2

For Phase 2, descriptive statistics for all determinant measures will be calculated. Items from the Mi-PARIHS will be plotted as originally described for visual inspection of the weakest domains. These statistics will be calculated by site and by stakeholder type to facilitate presentation to the CAB and to inform development of the implementation blueprint.

Phase 3 will not have a corresponding statistical analysis because it is entirely dedicated to the development of the site-specific blueprints.

#### Phase 4

##### Primary and secondary endpoint comparison

A Fisher's exact test will be conducted to evaluate the difference in proportions of GCS 13–15 TBI patients between Phase 2-enrolled participants (*n* = 140) and Phase 4-enrolled participants (*n* = 140). Another Fisher's exact test will also compare the difference in proportions of GCS 13–15 TBI patients with a negative POC whole blood TBI test who still received a head CT scan between Phase 2 and Phase 4. Subanalyses will be conducted for each site, as well.

##### Secondary outcome comparison

ED length of stay (in minutes) will be compared between patients who only received the POC whole blood TBI test and those who received the POC whole blood TBI test and a head CT scan using an analysis of covariance model including enrollment site as a covariate. This analysis will be conducted only for participants with a negative POC whole blood TBI test to control for clinical severity of the injury. Differences in these rates following implementation will be evaluated using the same approach (both for the overall cohorts and those with a negative POC whole blood TBI test only). An exploratory receiver operating characteristic AUC analysis will be conducted to differentiate patients who return to the ED for evaluation of the same injury within 2 weeks from those who do not, using whole blood GFAP and UCH-L1 as predictors.

To evaluate the effect of the implementation blueprint on perspectives regarding determinants and implementation outcomes, Mi-PARIHS domain scores will be compared pre- and post-implementation using repeated measures general linear model, including site as a covariate. Secondary comparisons will be made for implementation outcomes (e.g., AIM, FIM, and DCS).

## Discussion

This study protocol is the first documented hybrid type II effectiveness–implementation study to improve uptake of an objective blood biomarker test for patients with TBI in United States EDs. Our goal is to provide evidence that use of this test can reliably and safely improve clinical practice in EDs by reducing rates of unnecessary radiation exposure, decreasing time spent in the ED, and demonstrating efficacy of an implementation blueprint that can facilitate uptake of this test to meet these goals at similar Level 1 trauma center EDs.

The goals of this study have the potential for significant impact on public health. Researchers have recently suggested, assuming current CT scan trends continue, that cancers associated with repeated CT scans will eventually account for 5% of all new cancer diagnoses annually (28). Reducing CT scan rates for a population where <10% of patients are expected to have intracranial pathology can reduce unnecessary radiation exposure to the millions of mTBI patients evaluated annually in US EDs (1). Reducing length of stay in the ED has obvious benefits for triage efficiency and appropriate allocation of healthcare resources, while also enhancing patient health and wellbeing. A large retrospective cohort study of nearly 200 California hospitals found that patients admitted on high-crowding days experienced 5% greater odds of inpatient death and longer hospital length of stay overall, even after controlling for demographics, clinical complaints, comorbidities, and diagnoses (29). This suggests that prolonged time in the ED environment is associated with higher odds of negative outcomes. Boarding patients in the ED also increases risk for experiencing undesirable events, including missing a relevant medication and preventable adverse events (30). Implementation of the whole blood TBI test offers the opportunity to alleviate some of these concerns. It is important to note, however, that CT utilization rates can be impacted by many factors, including seasonal variation, time of day, changes in institutional policy, and clinician/personnel turnover, all of which could impact study results.

The literature in this area is sparse due to the relative novelty of using blood biomarkers for TBI assessment. S100B is a protein approved for the same use as the POC whole blood TBI test in Europe. A single-center study evaluated the use of S100B as an adjunct to the Scandinavian Neurotrauma Committee evaluation guideline for patients with minimal, mild, and moderate traumatic head injuries (17). The study found that one in five patient discharges were directly attributable to S100B testing, but compliance with the overall guideline was poor (17). Another study applied these guidelines, including S100B testing, to six emergency departments in the US (18). The authors found that the guidelines worked as intended and their application could have reduced CT usage by one-third. These studies correspond with a growing body of literature suggesting that clinical rules for ordering a head CT are effective but not applied broadly enough in clinical practice (31, 32). These results underscore the need for an implementation science approach, such as the one used in the present study, to actively identify barriers and facilitators to adoption of the POC whole blood TBI test in conjunction with clinical efficacy. One active study aims to compare the rate of head CT utilization between ED physicians blinded to the POC whole blood TBI test results and those who were unblinded to the POC whole blood TBI test results (19). Implementation science is not an explicitly stated method applied in that study's protocol, but the results should provide evidence complimentary to the present study.

### Anticipated impact on clinical practice

Several deliverables will be generated from the completion of this study that can meaningfully impact the clinical practice of evaluating mTBI patients in the ED. The primary deliverable is the open-access publication of site-specific implementation blueprints for designed to increase use of the POC whole blood TBI test. This will allow other Level 1 trauma center EDs with similar patient throughput and demographics to study how the test was implemented, enabling them to tailor strategies to at their own institutional contexts. We will also provide the first known evidence of the clinical utility of the test in predicting return visits to the ED for the same injury within a 2-week period. This protocol will also deliver crucial data on how implementation of the test reduces length of time spent in the ED, which is an endpoint with significant potential to influence uptake from a practical standpoint. Our intent is to use the data generated from the completion of this work to conduct a hybrid type III effectiveness–implementation clinical trial, evaluating the efficacy of optimized interventions to improve clinical uptake of the POC whole blood TBI test in EDs of varying sizes.
